# Clinical Efficacy of Pyrotinib Combined with Capecitabine in the Second-Line or Above Treatment for HER-2 Positive Advanced Breast Cancer and Its Association with Cell-Free DNA

**DOI:** 10.1155/2022/9449489

**Published:** 2022-10-07

**Authors:** Yajun Miao, Juan Chen, Rong Deng, Yufei Liu

**Affiliations:** ^1^Department of Oncology, The Second Affiliated Hospital of Nantong University, Nantong, China; ^2^Department of General Surgery, People's Hospital of Luhe District in Nanjing, Nanjing, China; ^3^Department of General Surgery, The Affiliated Cancer Hospital of Nanjing Medical University & Jiangsu Cancer Hospital & Jiangsu Institute of Cancer Research, Nanjing, China; ^4^Department of Medical Oncology, Jiangsu Cancer Hospital and Jiangsu Institute of Cancer Prevention and Treatment and The Affiliated Cancer Hospital of Nanjing Medical University, Nanjing, China

## Abstract

**Purpose:**

To evaluate the clinical efficacy of pyrotinib combined with capecitabine in the treatment of HER-2 positive breast cancer in real world and its correlation with cfDNA.

**Methods:**

From September 2020 to June 2021, 181 cases of HER-2 positive advanced breast cancer patients who were treated in Jiangsu Cancer Hospital and Nantong Cancer Hospital were analyzed. Patients were given pyrotinib combined with capecitabine or trastuzumab combined with capecitabine. Eighty-one patients who received pyrotinib+capecitabine second-line or above treatment were included in the pyrotinib group, and 100 patients who received trastuzumab+capecitabine second-line or above treatment were included in the trastuzumab group. The objective response rate (ORR) and clinical benefit rate (CBR) of the two groups were compared. The follow-up results of the patients were analyzed, and the progression-free survival (PFS) and adverse reactions were compared between the two groups. Plasma cfDNA was detected by real-time fluorescence quantitative PCR. The cfDNA levels of patients before and after treatment were compared, and the change of cfDNA levels in patients with different curative effects over time was recorded. The patients were further divided into high cfDNA expression and low cfDNA expression groups, and the PFS of patients with different cfDNA levels was analyzed. COX univariate and multivariate analysis of factors influencing posttreatment survival in patients with HER-2-positive breast cancer were performed.

**Results:**

The ORR of the pyrotinib group (58.02%) was significantly higher than that of the trastuzumab group (42.00%, *P* = 0.0369). Similarly, the CBR of the pyrotinib group (65.43%) was significantly higher than that of the trastuzumab group (49.00%, *P* = 0.0347). The incidence of adverse reactions between the two groups was not statistically significant (*P* > 0.05). The results of survival analysis showed that the PFS of the pyrotinib group was 8.02 ± 3.05 months, the PFS of the trastuzumab group was 7.11 ± 3.06 months, and the PFS of the pyrotinib group was significantly longer than that of the trastuzumab group (*P* = 0.035). The comparison of cfDNA levels between the two groups showed that on the 28th and 56th day of treatment, the cfDNA levels in the pyrotinib group were significantly lower than those in the trastuzumab group (*P* < 0.05). Long-term follow-up results showed that compared with patients with high cfDNA expression, the PFS of patients with low cfDNA expression was significantly prolonged (*P* < 0.05). The level of cfDNA is an independent risk factor affecting the prognosis of patients with HER-2-positive breast cancer.

**Conclusion:**

The combined use of pyrotinib and capecitabine has good clinical efficacy and high safety in patients with HER-2 positive breast cancer. The combined use of pyrotinib and capecitabine prolongs the PFS of patients and reduces the level of plasma cfDNA. Changes in cfDNA levels can reflect the therapeutic efficacy of patients with HER-2-positive breast cancer to a certain extent and can be used as a potential indicator for evaluating the prognosis of patients with HER-2-positive breast cancer.

## 1. Introduction

Breast cancer ranks first in the incidence of female malignant tumors. The incidence of breast cancer is on the rise, and the patients are showing a younger trend globally [1, 2]. Human epidermal growth factor receptor-2 (HER-2) overexpression is found in 15%-20% of breast cancer patients, with an aggressive phenotype. The prognosis of breast cancer patients is closely related to the overexpression and mutation of HER-2 [3]. HER-2-positive breast cancer patients have a higher risk of death. The standard treatment for advanced patients with HER-2-positive breast cancer is chemotherapy combined with targeted therapy, of which targeted therapy plays an important role. How to choose the appropriate targeted drugs is the key to improve the therapeutic effect [4]. As the first anti-HER-2 targeted therapy drug, trastuzumab has been clinically used for more than 10 years and has established its status as a first-line anti-HER-2 standard therapy, which can significantly improve the prognosis of patients with HER-2-positive breast cancer [5, 6] But the problems of drug resistance and toxicity in the treatment process have always plagued clinicians, and small molecule targeted drugs including imported lapatinib, neratinib, and domestically produced pyrotinib have appeared one after another. Pyrotinib belongs to a class of tyrosine kinase inhibitors that block HER-2 signaling by competing with intracellular adenosine triphosphate, thereby inhibiting the phosphorylation of protein tyrosine residues and blocking the transduction of downstream signaling pathways. To a certain extent, it can circumvent drug resistance to HER-2 antibodies and improve the therapeutic effect [7, 8]. Capecitabine is a new type of fluorouracil commonly used in clinic, which is absorbed by the small intestine and then enters the liver through the blood steam. The mechanism of inhibiting tumor cell proliferation is that it can complete the transformation to 5-fluorouracil (5-FU) in vivo. It prevents cancer cell mitosis and affects protein synthesis, and is often used in the treatment of metastatic breast cancer, especially for metastatic breast cancer that progresses after taxol or anthracycline chemotherapy [9].

Cell-free DNA (cfDNA) exists in peripheral blood and is a degraded endogenous DNA, and its expression level is closely related to pathophysiological processes of the body [10]. The cell-free DNA can not only show the whole picture of tumor genes but also indirectly reflect the occurrence and development of tumors to a certain extent [11]. Continuous detection and analysis of such tumor-derived cfDNA makes it possible to achieve noninvasive liquid biopsy, which brings important clinical value for tumor diagnosis, drug screening, prognosis assessment, and real-time treatment. A number of studies have shown that cfDNA is highly expressed in the peripheral blood of patients with lung cancer, colorectal cancer, breast cancer, esophageal cancer, and other malignant tumors and is related to tumor burden and prognosis [12–15].

The purpose of this study was to compare the efficacy of pyrotinib combined with capecitabine and trastuzumab combined with capecitabine and to investigate whether cfDNA can be used as a tumor marker for evaluating the efficacy of drug treatment. The study selected patients with HER-2 positive advanced breast cancer for comparative study, and the results are reported.

## 2. Material and Method

### 2.1. Case Data

This study is a prospective study. A total of 181 patients with HER-2-positive advanced breast cancer who were treated in Jiangsu Cancer Hospital and Nantong Cancer Hospital from September 2020 to June 2021 were selected. Patient's age ranged from 31 to 70 years old, with an average age (51.18 ± 9.80) years old. All patients were female. All patients and their families gave informed consent. Eighty-one patients who received pyrotinib plus capecitabine were included in the pyrotinib group, and 100 patients who received trastuzumab plus capecitabine were included in the trastuzumab group.

The inclusion criteria are as follows: [1] female patients with stage III-IV breast cancer who met the diagnostic criteria of Medical Oncology and were diagnosed by immunohistochemistry and pathology, [2] patients with immunohistochemistry (IHC) 2+ and primary or metastatic gene amplification by fluorescence in situ hybridization (FISH), and patients with IHC 3+ were defined as HER2 positive, [3] the patient's disease progressed after first-line chemotherapy, [4] there is at least one measurable lesion, [5] ECOG score ≤ 2, expected survival time ≥ 3 months, and [6] no psychological diseases, mental disorders, or nerve damage in the upper and lower limbs and have the ability to move independently.

The exclusion criteria are as follows: [1] patients with hypertension, [2] patients who have been treated with fluorouracil, [3] patients receiving other antineoplastic drugs during treatment, and [4] patients with other malignant tumors.

### 2.2. Diagnosis

#### 2.2.1. Immunohistochemistry (IHC) Detection

Immunohistochemical staining was performed with rabbit anti-human monoclonal anti-HER-2 (Maixin Biotechnology, Fuzhou, China, clone number: MXR001) using the Envision two-step procedure. The paraffin sections were dewaxed and dehydrated with xylene and alcohol (concentration from high to low), heated in ethylene diamine tetra-acetic acid (EDTA) water bath for 20 min, cooled to room temperature, and rinsed with PBS for 3 min × 3 times. Then put the sample in peroxidase blocker for 10 min at room temperature, rinse with PBS for 3 min × 3 times, dropwise add HER-2 primary antibody, incubate at room temperature for 60 min, rinse with PBS for 3 min × 3 times, and then add enzyme-labeled goat antibody. Mouse/rabbit IgG polymer IV, incubated at room temperature for 30 min, rinsed with PBS for 3 min × 3 times, then added 3-diaminobezidine (DAB) chromogenic solution, incubated at room temperature for 5 min, tap water to stop color development, hematoxylin stained for 2 min, tap water returned to blue, dehydrated with graded alcohol, transparent in xylene, and mounted with neutral gum.

The interpretation of results are as follows: HER-2 expression was divided into 0, 1+, 2+, 3+, and graded based on the proportion of positive cells and staining intensity: 0 if no staining or ≤10% of infiltrating cancer cells showed incomplete, weak cell membrane staining; 1+ if > 10% of infiltrating cancer cells showed incomplete, weak cell membrane staining; 2+ if >10% of infiltrating cancer cells showed weak-moderate intensity of intact cell membrane staining or ≤10% of infiltrating cancer cells show strong and complete cell membrane staining; 3+ if >10% of infiltrating cancer cells show strong, complete and uniform cell membrane staining. In order to ensure the accuracy of the interpretation of the results, two or more senior physicians in the pathology department performed repeated readings in a double-blind condition to determine the HER-2 staining results.

#### 2.2.2. Treatment

All patients completed various examinations after being enrolled in the group, and then were given capecitabine tablets (Xeloda, manufacturer: Shanghai Roche Pharmaceutical Co., Ltd., approved by Chinese medicine: H20073024) for treatment, orally administered within 30 minutes after meals, 1000 mg/m^2^, 2 times/d, 21 days as a cycle. On this basis, the trastuzumab group received intravenous trastuzumab on the first day of each cycle. The first dose was 8 mg·kg-1, and each subsequent dose was 6 mg·kg-1. While the pyrotinib group was given pyrotinib maleate (Ireni, manufacturer: Jiangsu Hengrui Pharmaceutical Co., Ltd., approved by Chinese medicine: H20180012), orally within 30 minutes after meals, 400 mg once, 1 time times/d, taking the medicine at the same time every day, 21 days as a cycle. Tumor evaluation was conducted for all patients before treatment, and efficacy evaluation was conducted every 6 weeks during treatment. The treatment was discontinued if the disease progressed on imaging or the adverse reaction reached grade III.

#### 2.2.3. Detection of Plasma cfDNA levels in Patients by Real-Time PCR

1. *Sample Collection and Pretreatment*. 2 m1 of venous blood from breast cancer patients (before and after treatment) were collected and placed in an EDTA anticoagulation tube for collection. Blood was centrifuged at 1900 × g at a low speed for 15 min as soon as possible (within 6 h). The plasma was placed in a clean microcentrifuge tube, diluted with PBS buffer, and centrifuged at 13 000 × g at a high speed. About 50 *μ*l of the supernatant was taken and used directly for cfDNA detection, or cryopreserved at -80°C
2. *Extraction of cfDNA from Plasma*. QIAamp DNA Blood Mini Kit (Qiagen Germany, 51104) was used, and the specific operations were carried out in strict accordance with the kit instructions
3. *Determination of cfDNA Level in Plasma*. Take 50 *μ*l of nucleic acid extract, and use QuantiDNA Direct cfDNA Test (DiaCarta) kit to determine the concentration of cfDNA. Primer 1 (97 bp): Forward: 5′-TGGCACATATACACCATGGAA-3′, Reverse: 5′-TGAGAATGATGGTTTC-3′, Primer 2 (300 bp): Forward: 5′-ACAACCTATTCCAAAATTGACCAC-3′, Reverse: 5′ -TTCCCTCTACACACTGCTTTGA-3′, cfDNA integrity index calculated as the ratio of LINE 300 bp and LINE 197 bp qRT-PCR results. The amplified fragment of the internal reference beta actin is 186 bp: the upstream primer of beta actin: 5′-TGGCACCCAGCACAATGAA-3′, and the downstream primer of beta actin: 5′-CTAAGTCATAGTCCGCCTAGAAGCA-3′.

### 2.3. Efficacy Evaluation

During chemotherapy, the patients were examined and evaluated by the same personnel every 6 weeks according to the RECIST1.1 criteria. The treatment response were divided into complete remission (CR, complete disappearance of all lesions), partial remission (PR, reduction in the sum of the diameters of lesions ≥ 30%), progressive disease (PD, the combined diameter of all target lesions increased by at least 20%, or the appearance of new lesions), and stable disease (SD, between PR and PD). Objective response rate (ORR) = (CR + PR)/number of cases; clinical benefit rate (CBR) is CR, PR, or SD, maintained for at least 6 months.

### 2.4. Adverse Reactions

The adverse reactions of the two groups of patients were accurately observed and recorded by the responsible nurse, and the adverse reactions were graded as I to V according to the evaluation criteria of common adverse events (National Cancer Institute-Common Terminology Criteria for Adverse Events version 4.0, NCI-CTCAE4.0). *Grade I:* mild; asymptomatic or mild; only clinical or diagnostic findings; no treatment required. *Grade II:* moderate; requires minor, topical, or noninvasive treatment; age-appropriate instrumental ADL. *Grade III:* severe or medically important, but not immediately life-threatening; leading to hospitalization or prolonged hospitalization; disability; limited self-care activities of daily living. *Grade IV:* life-threatening consequences; urgent medical attention required.

### 2.5. Follow-Up

Outpatient or telephone follow-up was used, and the two groups were followed up for at least 12 months after the end of treatment. The analysis was performed according to RECIST version 1.1, and the progression-free survival (PFS) of the two groups after treatment was accurately recorded (from randomization to patient time to tumor progression or death).

### 2.6. Statistical Methods

SPSS 21.0 statistical software was used to process data in this study. Measurement data were expressed as mean ± standard deviation. Independent samples were analyzed by Student's t test. Enumeration data were expressed as rates, and the *χ*^2^ test was used for analysis. The survival curve was drawn by log-rank test. The factors affecting the prognosis were screened out by COX univariate analysis, and then multivariate COX regression analysis was used to test whether multiple factors were related to survival. *P* < 0.05 was considered to be statistically significant.

## 3. Results

### 3.1. Baseline Data

Patients in both groups were females and the pathological type of cancers was invasive ductal carcinoma. *Trastuzumab Group:* age: 29-70 years old, mean age (50.91 ± 9.53) years old. *Pyrotinib Group:* 25-70 years old, mean age (49.46 ± 9.80) years old. There was no significant difference in clinicopathological data (age, menstrual status, ECOG score, hormone receptor status, metastatic site, drug resistance, previous treatment, and number of metastases) between the two groups of patients (*P* > 0.05), therefore patients in both groups were comparable (Table 1).

### 3.2. Detection of HER-2 Protein Expression by IHC

**Figure 1:**
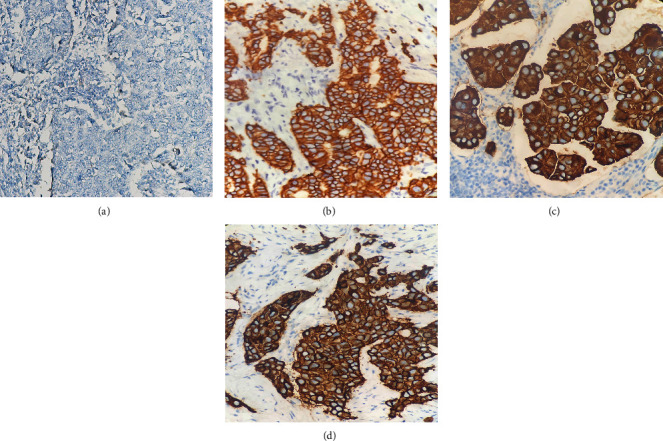
Expression of HER-2 protein in breast cancer by IHC (×20). (a) HER-2 protein expression is negative; (b)-(d) HER-2 protein expression is positive.

All tumors were evaluated by IHC. We randomly selected 3 cases of HER-2 positive patients. The IHC staining results showed that HER-2 is stained in the cell membrane and is brown. (Figure 1).

### 3.3. Clinical Efficacy

**Figure 2:**
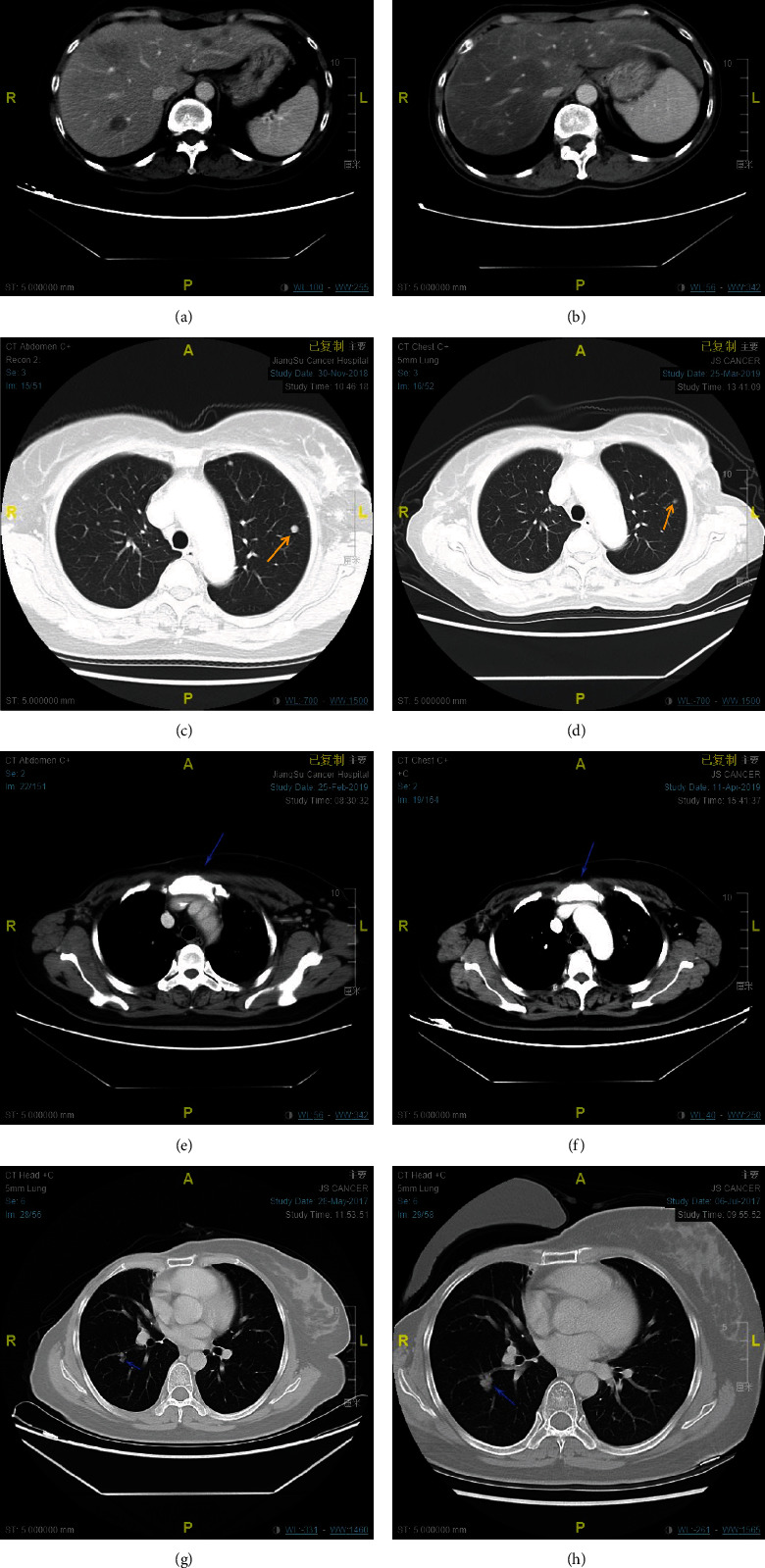
CT images of the patient before and after treatment. (a) CT images of patients with liver metastases diagnosed as CR before treatment; (b) CT images of patients with liver metastases diagnosed as CR after treatment with pyrotinib+capecitabine; (c) treatment of patients with pulmonary metastases diagnosed as PR CT images before treatment; (d) CT images of patients with pulmonary metastases diagnosed as PR after treatment with pyrotinib+capecitabine; (e) CT images of patients with pulmonary metastases diagnosed as SD before treatment; (f) CT images of patients with pulmonary metastases diagnosed as SD CT images of patients with pulmonary metastases treated with pyrotinib+capecitabine; (g) CT images of patients with pulmonary metastases diagnosed as PD before treatment; (h) CT images of patients with pulmonary metastases diagnosed as PD treated with pyrotinib+capecitabine CT images after the treatment.

The short-term efficacy evaluation results showed that the ORR of the pyrotinib group was 58.02%, including 6 cases of CR, 41 cases of PR, 28 cases of SD, and 10 cases of PD. The ORR of the trastuzumab group was 42.00%, of which 2 cases were CR. For example, 40 were PR, 39 were SD, and 15 were PD. The ORR was significantly higher in the pyrotinib group than in the trastuzumab group (*P* = 0.0369). The CT images of a patient who achieved different short-term effects in the pyrotinib group were selected for comparison before and after treatment (Figure 2). The results of long-term efficacy evaluation showed that the CBR of the pyrotinib group was 65.43%, which was significantly higher than that of the trastuzumab group (CBR = 49.00%, *P* = 0.0347) as shown in Table 2.

### 3.4. Adverse Reactions

The main adverse reactions in the two groups were diarrhea, nausea and vomiting, hypertension, stomatitis, leukopenia, and hand-foot syndrome. Most of the adverse reactions were grade I-II, and the incidence of grade III adverse reactions was low. The symptoms could be alleviated and improved after drug reduction or symptomatic treatment, and no grade IV adverse reactions occurred. There was no significant difference in the incidence of adverse reactions between the pyrotinib group and the trastuzumab group (all *P* > 0.05) as shown in Table 3.

#### 3.4.1. Progression-Free Survival (PFS)

**Figure 3:**
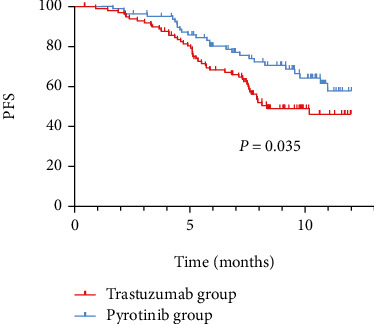
Kaplan-Meier curves of PFS in the two groups of patients.

The follow-up ended in June 2022. The survival of patients was grouped and analyzed according to drug resistance. The results showed that the median PFS in the trastuzumab group was 5.89 months, the median PFS in the pyrotinib group was 6.62 months, and the PFS in the trastuzumab group was 7.11 ± 3.06 months. PFS was 8.02 ± 3.05 months in the pyrotinib group. See Figure 3.

### 3.5. Changes in Plasma cfDNA levels Before and After Treatment

After treatment, the cfDNA levels decreased over time in both groups. After 28 days of treatment, the cfDNA of both groups was significantly lower than that before treatment (*P* < 0.05). At the same time, the results showed that on the 28th and 56th day of treatment, the cfDNA level of the patients in the pyrotinib group was significantly lower than that in the trastuzumab group (*P* < 0.05) (Table 4).

# *P* < 0.05, compared with before treatment; ^∗^*P* < 0.05, compared with pyrotinib group.

### 3.6. Analysis of cfDNA Expression Level and Patient PFS

**Figure 4:**
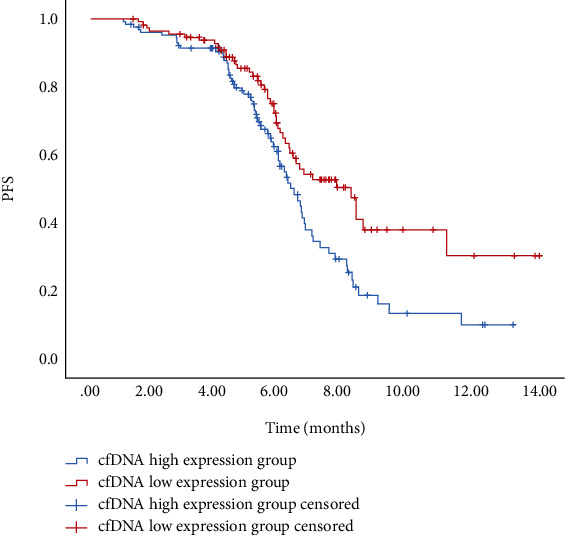
Kaplan-Meier curves of progression-free survival in patients with different cfDNA expression levels.

These 181 breast cancer patients were divided into groups according to cfDNA levels. With the median cfDNA value of 7.49 ± 0.97 ng/ml as the cut-off value, the patients with plasma cfDNA concentration higher than 7.49 ± 0.97 ng/ml were included in the cfDNA high expression group, and the plasma cfDNA concentration less than 7.49 ± 0.97 ng/ml were included in the cfDNA low expression group. The PFS of the two groups was analyzed. The results showed that the PFS of patients with low cfDNA expression was 8.12 ± 6.35 months, and the PFS of patients with high cfDNA expression was 6.35 ± 5.78 months. Compared the patients with high cfDNA expression, the PFS of patients with low cfDNA expression was significantly prolonged (*P* < 0.05) as shown in Figure 4.

### 3.7. Cox Univariate and Multivariate Analysis

The age, hormone receptor expression, ECOG, metastasis, cfDNA expression, drug resistance, previous treatment, number of metastases, and other indicators of 181 breast cancer patients were subjected to COX univariate and multivariate regression analysis. The results showed that ECOG (*P* = 0.009) and cfDNA level (*P* = 0.003) were clinical factors affecting the prognosis of breast cancer patients. Further COX multivariate analysis found that cfDNA level (*P* = 0.018) was an independent risk affecting the prognosis of breast cancer patients factor (Table 5 and Table 6).

## 4. Discussion

Pyrotinib is a small-molecule, irreversible tyrosine kinase inhibitor that acts on the three targets of HER-1, HER-2, and HER-4. It has a small molecular mass and can be administered orally. Pyrotinib binds covalently to the ATP-binding sites of the kinase domains of intracellular epidermal growth factor receptor and HER-2, preventing formation of intracellular homo- and heterodimerization of epidermal growth factor receptor and HER-2 in tumor cells, thereby inhibiting its autophosphorylation. This mechanism in turn blocks the activation of downstream signaling pathways and inhibits tumor cell growth. The antitumor effect of pyrotinib has been clinically confirmed [16]. A phase I clinical trial conducted by the Cancer Hospital, Chinese Academy of Medical Sciences confirmed the safety of single-agent pyrotinib in the treatment of solid tumors and had a high tumor shrinkage rate [17]. A phase II clinical study compared the safety and efficacy of pyrotinib combined with capecitabine and lapatinib combined with capecitabine in the treatment of HER-2 positive metastatic breast cancer. The results showed that the overall response rate and progression-free survival of pyrotinib+capecitabine were better than lapatinib in patients with HER-2-positive breast cancer who had failed previous taxane, anthracycline and/or trastuzumab therapy Ni+capecitabine [18]. Since pyrotinib was launched in China in August 2018, there have been few reports on the clinical application of pyrotinib in the real world. This study analyzed the efficacy and adverse reactions of pyrotinib combined with capecitabine compared with trastuzumab combined with capecitabine and discussed the effect and safety of pyrotinib combined with capecitabine in HER-2 positive breast cancer patients. Meanwhile, we compared the differences in cfDNA levels of patients before and after treatment, and further divided patients into cfDNA high expression group and cfDNA low expression group according to cfDNA expression level. This study analyzed the factors affecting the survival rate of patients with HER-2 positive breast cancer and provided scientific data for helping clinical treatment plan and prognosis prediction.

Related studies have found that pyrotinib plus capecitabine is well tolerated in the majority of HER2-naïve populations and shows promising antitumor activity in patients with HER2-positive metastatic breast cancer [19]. The results of this study showed that the ORR and CBR of the pyrotinib group were significantly higher than those of the trastuzumab group, and the PFS of the pyrotinib group was prolonged compared with that of the trastuzumab group, indicating that the combination of pyrotinib and capecitabine positive have better clinical outcomes and higher survival benefits for HER-2 positive breast cancer patients. The main adverse reactions in the two groups were diarrhea, nausea and vomiting, hypertension, stomatitis, leukopenia, and hand-foot syndrome. The incidence of adverse reactions in the two groups was similar, indicating that pyrotinib combined with capecitabine did not seriously aggravate the adverse reactions of patients. Symptoms can be alleviated and improved after drug reduction or symptomatic treatment. For HER-2-positive metastatic breast cancer, the standard first-line anti-HER-2 therapy based on trastuzumab has been established, but changes in HER-2 molecular spatial structure and downstream signaling pathways may lead to trastuzumab resistance. Due to its different mechanism of action, pyrotinib has shown advantages in the treatment of various aspects of second-line anti-HER-2. At the same time, pyrotinib has the advantages of convenient administration and less toxic and side effects. Moreover, it is effective for patients who are resistant to anthracyclines, taxanes, and trastuzumab. Combined with the current unavailability of trastuzumab emtansine in China and the medical insurance policy of related targeted drugs, it provides an effective treatment for patients with metastatic breast cancer, who are not tolerated to intravenous chemotherapy.

cfDNA is a general term for free DNA in peripheral blood, which often exists in the form of protein complexes, including tumor-derived DNA and normal cell-derived DNA [11]. Due to the reduced nuclease activity in the plasma of cancer patients, the clearance rate of cfDNA is reduced, resulting in higher cfDNA concentrations in cancer patients than in healthy individuals [15, 20]. Therefore, detecting the level of cfDNA can monitor the condition of cancer tissue in real time. In this study, the plasma cfDNA concentrations of patients before and after treatment were detected. The results showed that the cfDNA concentrations of the two groups of patients after 28 days of treatment were significantly lower than those before treatment, and the cfDNA levels in the pyrotinib group were significantly lower than those in the trastuzumab group. Long-term dynamic monitoring can evaluate the curative effect of patients and serve as a curative effect evaluation index for patient prognosis. The patients were further divided into cfDNA high expression group and low expression group according to the concentration of cfDNA, and it was found that the PFS of patients with low cfDNA expression was significantly prolonged compared with that of patients with high cfDNA expression. Moreover, the results of COX univariate analysis and multivariate analysis showed that cfDNA was one of the independent risk factors affecting the prognosis of patients with HER-2 positive breast cancer.

In conclusion, compared with trastuzumab combined with capecitabine, pyrotinib combined with capecitabine has higher clinical efficacy and high safety, can prolong the PFS of patients, and reduce the level of plasma cfDNA in patients.

The change of cfDNA level can reflect the efficacy of pyrotinib combined with capecitabine in the treatment of patients with HER-2 positive breast cancer to a certain extent, which can be used as a potential indicator to evaluate the prognosis of patients with HER-2 positive breast cancer, which is worthy of further clinical research and promotion.

## Figures and Tables

**Table 1:** Baseline characteristics of 181patients.

Item	Trastuzumab group	Pyrotinib group	*P*
Age	50.91 ± 9.53	49.46 ± 9.80	0.186
Menstrual status			
Premenopausal	22	21	0.5997
Menopause	78	60	
ECOG			
0-1	85	65	0.4321
2	15	16	
Hormone receptor status			
ER and/or PR positive	77	59	0.6045
ER and/or PR negative	23	22	
Metastasis			
Lung	19	13	0.5286
Liver	10	4	
Nonvisceral metastasis	41	35	
Multiple metastasis	30	29	
Number of metastases			
1	68	46	0.1255
≥ 2	32	35	
Tumor size			
> 5 cm	34	20	0.1938
≤ 5 cm	66	61	
Clinical staging			
III	39	41	0.1337
IV	61	40	

**Table 2:** Comparison of clinical efficacy between the two groups of patients.

Group	CR	PR	SD	PD	ORR (*n*, %)	CBR (*n*, %)
Trastuzumab group	2	40	39	15	42 (42.00)	49 (49.00)
Pyrotinib group	6	41	28	10	47 (58.02)	53 (65.43)
p					0.0369	0.0347

**Table 3:** Comparison of adverse reactions between the two groups of patients (*n*).

Adverse reaction	Pyrotinib group	Trastuzumab group	*P*
Diarrhea			
I-II	53	73	0.8176
III	10	12	
Nausea and vomiting			
I-II	10	13	0.6389
III	3	2	
Hypertension			
I-II	15	21	1.000
III	1	1	
Stomatitis			
I-II	10	16	1.000
III	1	2	
Leukopenia			
I-II	29	42	0.6444
III	3	2	
Hand-foot syndrome			
I-II	6	5	0.6044
III	3	1	

**Table 4:** Changes of cfDNA levels in the two groups of patients before and after treatment (ng/ml).

Time	Trastuzumab group	Pyrotinib group
Before treatment	24.17 ± 7.85	23.22 ± 8.96
D7 after treatment	22.26 ± 6.38	21.59 ± 5.53
D28 after treatment	15.69 ± 4.51^∗^^#^	12.78 ± 3.46^#^
D56 after treatment	11.08 ± 1.49^∗^^#^	9.35 ± 1.32^#^

**Table 5:** COX Univariate Analysis.

Factor	B	SE	Wald	Df	Sig.	Exp	95.0% CI for Exp (B)	
							Lower	Upper
Age	-0.646	0.651	0.986	1	0.321	0.524	0.146	1.876
Hormone receptor expression	1.291	0.509	1.025	1	0.116	0.462	0.941	1.695
Metastasis	-0.933	0.722	1.667	1	0.197	0.393	0.096	1.621
ECOG	1.668	0.636	6.891	1	0.009	5.303	1.526	18.43
cfDNA expression	2.308	0.788	8.582	1	0.003	10.06	2.147	47.10
Number of metastases	0.214	0.323	0.44	1	0.507	1.239	0.658	2.334

**Table 6:** COX Multivariate Analysis.

Factor	B	SE	Wald	Df	Sig.	Exp	95.0% CI for Exp (B)	
							Lower	Upper
cfDNA expression	4.201	1.768	5.645	1	0.018	66.76	2.087	2136
ECOG	-1.312	1.048	1.689	1	0.194	0.273	0.038	1.936

## Data Availability

All experimental data used to support the findings of this study are available from the corresponding author upon request.
